# MCLCBA: multi-view contrastive learning network for RNA methylation site prediction

**DOI:** 10.1186/s12859-025-06306-x

**Published:** 2025-11-19

**Authors:** Honglei Wang, Xuesong Zhang, Yanjing Sun, Zhaoyang Liu, Lin Zhang

**Affiliations:** 1 School of Information and Control Engineering, China University of Mining and Technology, Xuzhou, 221116 China; 2 School of Computer Science and Technology, China University of Mining and Technology, Xuzhou, 221116 China; 3 School of Information Engineering, Xuzhou College of Industrial Technology, Xuzhou, 221400 China; 4 School of Information Engineering (School of Big Data), Xuzhou University of Technology, Xuzhou, 221018 China

**Keywords:** RNA methylation, Site prediction, Deep learning, Attention mechanism, Contrastive learning

## Abstract

**Background:**

RNA methylation (RM) regulates gene expression regulation, RNA stability, and protein translation. Accurate prediction of RM modification sites is essential for understanding their biological functions. However, existing wet-lab detection techniques face challenges including operational complexity and high costs. Deep learning (DL) methods have been applied to this task. However, existing methods show performance degradation with smaller training datasets. For instance, the Bidirectional Gated Recurrent Unit (BGRU) demonstrates substantial performance degradation. Contrastive Learning Network (CNN) can extract local pattern features but learns overly specific patterns with sample-limited data, resulting in poor feature generalization. Bidirectional Long Short-Term Memory (BiLSTM) excels at modeling long-range dependencies but cannot sufficiently learn gating mechanism parameters to capture effective sequence representations with limited samples. Transformer processes sequences in parallel and captures global dependencies through self-attention, but its quadratic computational complexity and large parameter count make it prone to overfitting on small datasets. Current DL methods show reduced performance when training data is limited.

**Results:**

This study proposes a Multi-view Contrastive Learning with CNN-BiLSTM-Attention (MCLCBA) framework for RM modification site prediction. The multi-view approach comprises a primary view and auxiliary view, where the primary view utilizes DNA Bidirectional Encoder Representations from Transformers (DNABERT) to extract sequence contextual features, and the auxiliary view employs Chaos Game Representation (CGR) to extract structural features. Feature extraction includes four components: data augmentation, multi-view encoders, projection heads, and contrastive loss functions. By implementing dual differential data augmentation strategies and constructing multi-view network architectures for feature processing and fusion, the model learns discriminative feature representations invariant to data augmentation through maximizing positive sample similarity while minimizing negative sample similarity. This effectively addresses sample-limited feature learning scenarios. Experimental results on the sample-limited m^7^G dataset demonstrate that MCLCBA achieves AUROC and AUPRC of 85.64% and 86.94%, respectively, improving upon existing methods by 5–6% in both metrics.

**Conclusions:**

Through multi-view contrastive learning, MCLCBA provides an approach for RM sites under sample-limited scenarios.

## Background

Recent epitranscriptomic research has revealed that RNA modifications are widespread in the transcriptome and regulate gene expression alongside epigenetic mechanisms. As of 2024, the latest version of the RNA modification database Modomics has catalogued over 170 types of RNA modifications, with RNA methylation (RM) modifications accounting for approximately 80% of all RNA modification types, with methylation being the most prevalent modification type [1]. RM refers to the biochemical process of adding methyl groups to specific nucleotides in RNA molecules, primarily occurring on various types of RNA molecules including messenger RNA (mRNA), ribosomal RNA (rRNA), transfer RNA (tRNA), long non-coding RNA (lncRNA), small nuclear RNA (snRNA), microRNA (miRNA), and circular RNA (circRNA) [2]. Common RM modifications include: N1-methyladenosine (m^1^A), 5-methylcytosine (m^5^C), 5-methyluridine (m^5^U), 2′-O-methyladenosine (Am), 2′-O-methylcytidine (Cm), 2′-O-methylguanosine (Gm), 2′-O-methyluridine (Um), N6-methyladenosine (m^6^A), N7-methylguanine (m^7^G), and N6,2′-O-dimethyl adenosine (m^6^Am). Based on the chemical properties of modification sites, these modifications can be categorized into base RM modifications (such as m^6^A, m^1^A, and m^5^C) and ribose RM modifications (such as Am, Cm, Gm, and Um) [3]. From a functional classification perspective, RM modifications affect RNA stability, translation efficiency, localization, and structure [4].

RM regulates RNA transcription, splicing, export, folding, maturation, translation, and stability throughout the RNA lifecycle, thereby modulating gene expression and cellular signaling. With the continuous development of high-throughput sequencing approaches and bioinformatics analysis methods, our understanding of RNA modification types, distribution patterns, and functions continues to deepen. RNA modifications are reversible and respond to environmental and developmental signals [5].

High-throughput sequencing methods enable researchers to identify different types of RNA modifications experimentally [6–10]. Although experimental methods provide valuable information about the locations of RNA modifications in different species, traditional wet-lab experimental techniques are time-consuming and labor-intensive [11, 12]. In this context, sequence-based computational prediction methods complement experimental methods [13–19]. Traditional computational prediction methods typically rely on feature extraction methods derived from human understanding. The method first employs manually crafted features such as nucleotide composition [20], nucleotide frequency [21], nucleotide density [22], and nucleotide chemical property and frequency [23], etc., followed by machine learning (ML) classifiers such as support vector machine [24], random forest [25], and extreme gradient boosting [26], etc., for prediction. However, the manually crafted features used in ML methods require a high degree of prior knowledge and may contain redundant information.

Deep learning (DL) methods have improved prediction accuracy, particularly when combined with natural language processing (NLP) techniques. DL is now widely used for predicting RM modification sites. The combination of DL and NLP improves of RM site prediction, but this performance improvement correlates with training sample size. According to statistical learning theory, the generalization ability of DL models follows the sample complexity bounds, meaning there is a proportional relationship between the number of model parameters and the number of training samples required. Under limited sample size conditions, models tend to memorize specific features of the training data rather than learning generalizable biological patterns, leading to performance degradation on independent test sets. For example, Rajput et al. pointed out that model training with limited sample sizes leads to insufficient research, increasing the risk of model overfitting [27]. Huang et al.’s BERMP method showed that the bidirectional gated recurrent unit (BGRU) performed inconsistently on datasets of different sizes. Specifically, under conditions of limited sample size, BGRU does not sufficiently learn m^6^A features in Saccharomyces cerevisiae, and its results are inferior to those of traditional ML approaches [28]. As shown in Table 1, the training set for the Saccharomyces cerevisiae species in BERMP contains only 1100 positive samples, and the test set contains only 207 positive samples, far below the tens of thousands of positive samples for other species in BERMP. Under the same predictor, only for Saccharomyces cerevisiae does the BGRU yield significantly lower prediction metrics than traditional ML methods, thereby exposing the limitations of DL combined with NLP in feature learning under limited sample size conditions. Through careful analysis of the BGRU model’s insufficient feature learning from both feature representation and model construction perspectives, the following issues are identified: First, incomplete feature representation: RNA word embedding (RNAWE) under limited sample size conditions cannot provide sufficient contextual information for DL models to construct complete feature representations of RNA methylation sites. The limited training samples result in sparse and inadequate word vector representations, failing to capture the complex sequence patterns essential for accurate methylation site identification. Second, limited pattern recognition capability of the constructed model: The BGRU exhibits constrained sequence pattern recognition capabilities under limited sample size conditions. Although BGRU can capture sequence dependencies bidirectionally, the insufficient training data leads to inadequate parameter learning in its gating mechanisms, making it difficult to form effective sequence feature representations. Consequently, BGRU’s predictive performance is highly sensitive to data scale and shows significant degradation when sample size is insufficient. The Gene2vec proposed by Zou et al. was the first to introduce the Word2vec embedding model into gene sequence learning, providing new insights for predicting RM modification sites. This method employs four encoding strategies: One-hot encoding, Neighboring Methylation State Encoding (NMSE), RNAWE, and Word2vec. Correspondingly, the authors designed four distinct convolutional neural networks (CNNs) for feature extraction and integrated prediction [29]. However, although CNN can effectively extract local pattern features from sequences, under limited sample size conditions it tends to learn overly specific local patterns, leading to inadequate feature generalization capability. Zhang et al. proposed the EDLm6Apred, which combines One-hot encoding, RNAWE, and Word2vec feature encoding with bidirectional long short-term memory (BiLSTM) to form an ensemble DL framework for human m^6^A site prediction [30]. While BiLSTM excels at capturing long-range dependencies, under limited sample size conditions the parameter learning of its gating mechanisms becomes insufficient, making it difficult to form effective sequence feature representations.

**Table 1:** Prediction methods of RM modification sites base on DL

Tool	Modification	Number of positive in train set	Number of positive in test set	Sample length(nt)	Encoding scheme(s)	Algorithm	Species
BERMP	m^6^A	1100	207	51	ENAC; RNAWE	RF; BGRU	*S. cerevisiae*
		2100	418	101			*A. thaliana*
		53,405	13,351	301			Mammalia
		45,218	11,305	191			Mammalia
Gene2vec	m^6^A	45,052	11,687	1001	One-hot; NMSE; RNAWE; Word2vec	CNN	*H. sapiens* *M. musculus*
		–	5228				*H. sapiens*
		44,289	8020				*H. sapiens* *M. musculus*
EDLm6Apred	m^6^A	44,898	8635	1001	One-hot; RNAWE; Word2vec	BiLSTM	*H. sapiens*
DeepM6ASeq	m^6^A	24,532	6307	101	One-hot	CNN + BiLSTM	*H. sapiens*
		18,862	4701				*M. musculus*
		11,055	2826				Zebrafish
Plant6mA	m^6^A	29,237	7300	41	AE	Transformer	Rosaceae
			31,873				*A. thaliana*
MSCAN	m^6^A	41,307	5901	21; 31; 41	Word2vec	self- and cross-attention	*H. sapiens*
	m^1^A	7357	1051				
	m^6^Am	1172	167				
	m^5^C	5953	850				
	m^5^U	863	123				
	m^7^G	605	86				
	Am	848	121				
	Cm	1058	151				
	Gm	636	90				
	Um	1438	205				
	I	5164	737				
	Ψ	1989	284				
CR-NSSD	m^6^A	1307	–	51	CGR	CRE	*S. cerevisiae*
		1130	–	41			*H. sapiens*
		725	–	41			*M. musculus*
BERT2OME	Nm	215	46	41	BERT	CNN	*H. sapiens1*
		499	–				*H. sapiens2*
		89	–				*S. cerevisiae*
		10	–				*M. musculus*
MRM-BERT	m^6^A	–	–	101	DNABERT; EIIP; BE; NCP; ENAC; KMFE; CKSNAP	CNN	*H. sapiens*
	m^1^A						
	m^6^Am						
	m^5^C						
	m^5^U						
	m^7^G						
	Am						
	Cm						
	Gm						
	Um						
	I						
	Ψ						

In recent years, the Transformer architecture and its attention mechanism have have been applied for RM site prediction. Shi et al.’s Plant6mA uses an adaptive embedding (AE) lookup table to encode nucleotide sequences and position information jointly, and employs a lightweight Transformer encoder with an 8-head attention mechanism to capture multi-scale correlations within sequences, enabling the prediction of m^6^A sites in Rosaceae and Arabidopsis [31]. Wang et al.’s MSCAN uses multi-scale self-attention and cross-attention mechanisms. This model constructs three different scale subsequences of 21-nt, 31-nt, and 41-nt centered on RM sites, combining Word2vec encoding with multi-scale self-attention and cross-attention fusion networks [32]. The model employs the self-attention mechanism to capture intra-sequence dependencies and the cross-attention mechanism to establish feature associations across different scales, achieving multi-level fusion and synergistic enhancement of cross-scale features through feature fusion, improving upon Transformer encoders. Li et al.’s CR-NSSD employs Chaos Game Representation (CGR) theory to integrate positional information of RNA sequences into single nucleotide states for constructing pre-encoded representations [33]. Through a Cross-domain Reconstruction Encoder (CRE), the method simultaneously learns both sequential dependencies and structural dependencies among nucleotides. Sequential dependencies are captured through three-layer causal dilated CNN feature extraction, while structural dependencies are learned by constructing self-correlation graphs through regional self-attention mechanisms and computing Laplacian operators in the spectral domain. The method then projects to the frequency domain via discrete Fourier transform to capture common patterns of sequence and structural dependencies. Finally, through masked attention mechanisms to reduce redundant information and combining reconstruction loss with binary cross-entropy loss for model training, the method achieves m^6^A site prediction for Saccharomyces cerevisiae, humans, and mice. CGR, as a fractal geometry-based sequence representation method, has been applied in biological sequence analysis. The above three methods utilize Transformers to process sequence information in parallel and capture global dependencies through self-attention or cross-attention mechanisms. However, their quadratic computational complexity and large number of parameters make the models prone to overfitting on datasets with limited sample sizes.

Large language models (LLMs), the Bidirectional Encoder Representations from Transformers (BERT) framework, based on Transformer architecture [34], can address insufficient feature learning in limited-sample datasets. The BERT2OME proposed by Soylu et al. applied this approach. This method combines a pre-trained BERT model with a CNN to predict Nm modification sites under the condition of only 215 positive samples [35]. By leveraging the features learned by BERT on large-scale DNA sequence data, the method achieves good predictive performance on limited Nm annotated data, showing that pre-trained language models in addressing the issue of insufficient feature learning in scenarios with limited sample sizes. Furthermore, to extend this technology to nucleic acid sequence analysis, the DNA Bidirectional Encoder Representations from Transformers (DNABERT) model, based on Transformers, learned general statistical patterns and combination patterns of nucleotide sequences through unsupervised pre-training on a large-scale DNA sequence corpus [36]. Considering the high similarity between RNA and DNA in sequence composition, the similarity of nucleotide triplet combinatorial patterns exceeds 95%, with differences only in thymine-uracil substitutions. The combinatorial patterns and statistical features of their nucleotide sequences exhibit strong consistency. Therefore, based on this sequence feature similarity, the pre-trained representations of DNABERT can effectively transfer to RNA modification prediction tasks [36, 37]. The MRM-BERT proposed by Wang et al. fine-tunes the DNABERT pre-trained model and employs a CNN to extract six feature encodings: Electron–Ion Interaction Pseudopotential (EIIP), Nucleotide Chemical Property (NCP), Enzyme-Nucleic Acid Interaction (ENAC), Binary Encoding (BE), Composition of k-spaced Nucleotide Pairs (CKSNAP), and k-mer Frequency Encoding (KMFE). Features from the CNN and BERT modules fuse at the fully connected layer to construct a hybrid DL framework, enabling precise prediction of 12 common human RNA modifications (including m^6^A, m^5^C, and m^1^A) [38]. This framework leverages complementary information between DNABERT representations and traditional features, demonstrating the efficacy of pre-training-fine-tuning strategies for feature learning in limited-sample scenarios.

However, all aforementioned models adopt supervised learning paradigms, lacking effective deep exploitation of labeled data. This limitation constrains their applicability under limited sample size conditions. We apply contrastive learning for RM site prediction, enhancing generalization capability through structured representation learning from scarce labels. Contrastive learning is a self-supervised representation learning approach. The Simple Framework for Contrastive Learning of Visual Representations (SimCLR), introduced by Chen et al., learns invariant representations through data augmentation and contrastive loss optimization [39]. Chen et al. showed that a contrastive learning framework with carefully designed augmentations could achieve competitive results without requiring specialized architectures. The model operates by maximizing feature similarity between positive pairs (augmented views of identical instances) while minimizing similarity between negative pairs (distinct instances).

**Fig. 1:**
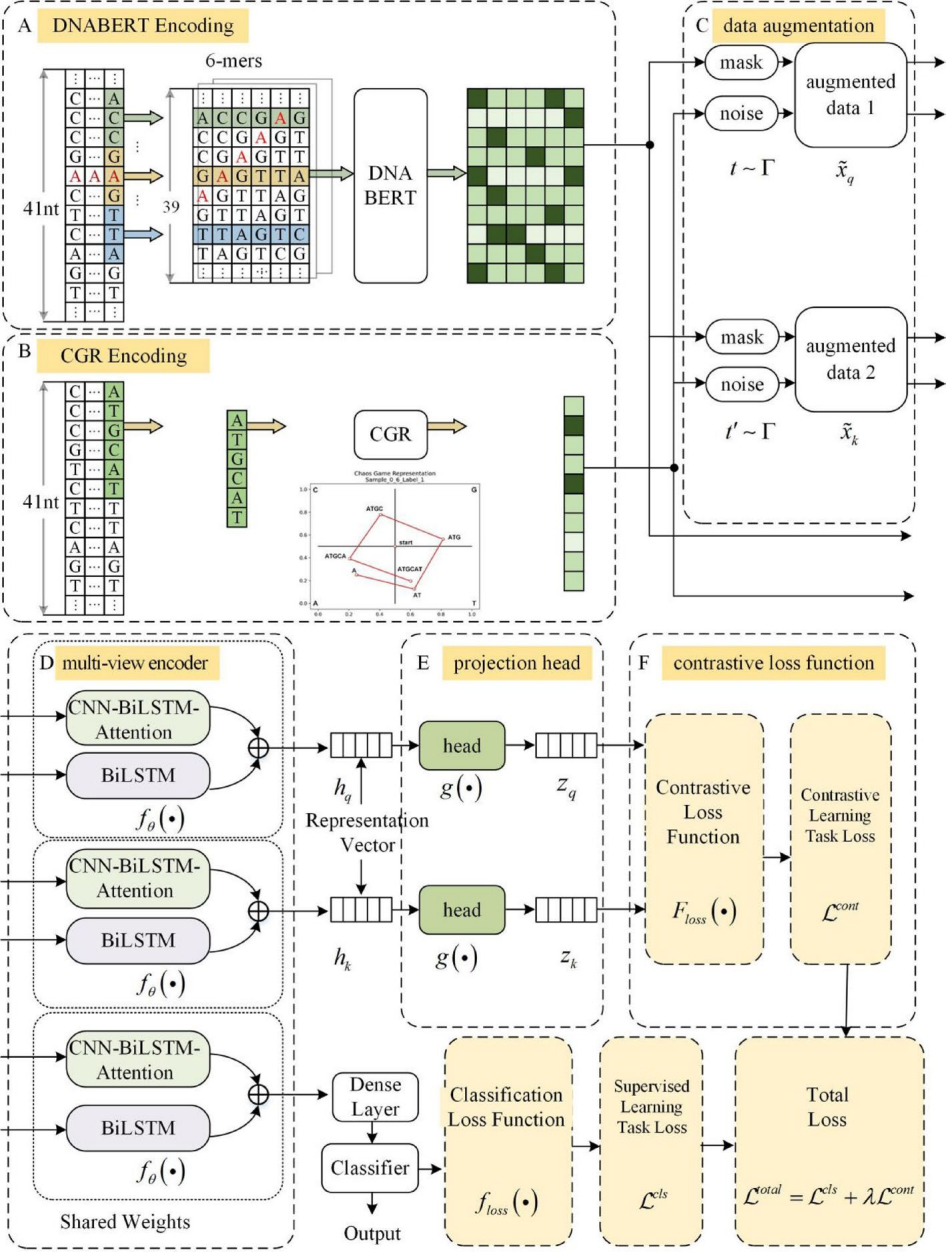
Model structure diagram of MCLCBA. **A** DNABERT module; **B** CGR module; **C** Data augmentation. **D** Multi-view encoder. **E** Projection head. **F** Contrastive loss function

In this context, this paper proposes a multi-view contrastive learning network based on CNN, BiLSTM and attention mechanism for RM site prediction (MCLCBA). This framework incorporates a contrastive learning paradigm to extract discriminative universal features from labeled RNA sequences by maximizing representational similarity within homogeneous samples while minimizing similarity across heterogeneous samples. This approach improves model generalization under sample-limited conditions. At the feature representation level, MCLCBA employs a dual-branch architecture for comprehensive feature extraction. Branch 1 processes DNABERT-encoded sequential representations through a hybrid CNN-BiLSTM-Attention module, where the CNN layer captures local sequence motifs, BiLSTM models bidirectional dependencies, and the attention mechanism identifies critical positions for modification sites. Branch 2 independently processes CGR-encoded structural features through a dedicated BiLSTM network that captures chaos game representation patterns reflecting sequence composition and structure. Critically, CGR provides unique geometric and fractal-based signals that cannot be captured by the DNABERT + CNN-BiLSTM-Attention pipeline alone. These parallel branches extract complementary features from sequential and structural perspectives respectively, which are subsequently aligned through projection heads and optimized via contrastive learning to maximize inter-view consistency while maintaining discriminative power. The complete architecture of MCLCBA is illustrated in Fig. 1.

## Results

### Evaluation metrics

To quantify predictive performance comprehensively, we adopt standardized metrics:

Sensitivity (Sn; Recall), Specificity (Sp), Accuracy (Acc), Precision (Pre), F1-score (F1), Matthews Correlation Coefficient (MCC), Receiver Operating Characteristic (ROC) curve with Area Under the Curve (AUCROC), and Precision–Recall (PR) curve with Area Under the Curve (AUCPR). Mathematical definitions follow.

1 $$ {\text{Recall}} = {\text{Sn}} = \frac{{{\text{TP}}}}{{{\text{TP}} + {\text{FN}}}} $$

2 $$ {\text{Sp}} = \frac{{{\text{TN}}}}{{{\text{TN}} + {\text{FP}}}} $$

3 $$ {\text{Acc}} = \frac{{{\text{TP}} + {\text{TN}}}}{{{\text{TP}} + {\text{TN}} + {\text{FP}} + {\text{FN}}}} $$

4 $$ {\text{Pre}} = \frac{{{\text{TP}}}}{{{\text{TP}} + {\text{FP}}}} $$

5 $$ {\text{F}}1 = 2 \times \frac{{{\text{Precision}} \times {\text{Recall}}}}{{{\text{Precision}} + {\text{Recall}}}} $$

6 $$ {\text{MCC}} = \frac{{{\text{TP}} \times {\text{TN}} - {\text{FP}} \times {\text{FN}}}}{{\sqrt {({\text{TP}} + {\text{FP}}) \times ({\text{TP}} + {\text{FN}}) \times ({\text{TN}} + {\text{FP}}) \times ({\text{TN}} + {\text{FN}})} }} $$

where TP, TN, FP, and FN denote the number of true positives, true negatives, false positives, and false negatives, respectively. All metrics range over [0, 1] except MCC ∈ [− 1, 1]. Higher values correspond to improved predictive performance across all metrics.

## Results analysis

To assess MCLCBA components, this study conducted ablation experiments based on the m^7^G dataset using fivefold cross-validation.

We tested four DNABERT variants (k = 3, 4, 5, 6) as CNN inputs. As Table 2 shows, DNABERT-6 achieved the highest performance, with AUROC = 0.7934 and AUPRC = 0.7902. Therefore, the results in this paper are based on the pre-trained model with k = 6.

**Table 2:** Performance comparison of four DNABERT variants

Variants	AUROC	Acc (%)	Sn (%)	Sp (%)	MCC (%)	Pre (%)	F1 (%)	AUPRC
DNABERT-3	0.7932	71.9	73.39	71.27	44.55	70.41	72.31	0.7808
DNABERT-4	0.7847	70.33	**80.66**	66.85	41.69	60.0	**73.11**	0.7648
DNABERT-5	0.7899	70.74	73.22	69.76	42.21	68.26	71.45	0.7718
DNABERT-6	**0.7934**	**75.39**	72.31	**77.08**	**44.55**	**73.1**	72.37	**0.7902**

Bold values represent the best results

### Comparison with other different learning models

Ablation study on branch 1 architecture. Using DNABERT-6 encoding, we tested three branch 1 architectures: CNN + BiLSTM + Attention(CBA), CNN, and CNN-BiLSTM. As shown in Table 3, performance improved with model complexity, and the CBA performed best. CNN-BiLSTM improved AUROC by 1.59% and AUPRC by 1.29% relative to CNN. BiLSTM layers captured bidirectional context effectively. Furthermore, CBA exhibited additional gains over CNN-BiLSTM, with 0.12% AUROC increase and 0.10% AUPRC improvement, accompanied by substantial enhancements in all six auxiliary metrics. The attention mechanism helped identify relevant sequence regions.

**Table 3:** Ablation study results for branch 1 architecture

Classifiers	AUROC	Acc (%)	Sn (%)	Sp (%)	MCC (%)	Pre (%)	F1 (%)	AUPRC
CNN	0.7934	**75.39**	72.31	77.08	44.55	73.1	72.37	0.7902
CNN + BiLSTM	0.8093	72.13	72.31	72.12	44.59	72.35	72.33	0.8031
CBA	**0.8105**	74.26	**74.21**	**78.11**	**48.68**	**73.58**	**73.83**	**0.8041**

Bold values represent the best results

### Comparison of branch 2 architectures

Integration of branch 2 with optimized branch 1. We then added branch 2 to the CBA architecture. To determine the optimal processing method for CGR features in branch 2, we compared two configurations while keeping branch 1 fixed. We applied both CNN and BiLSTM networks to extract features from CGR encoding, designated as CBA + CNN and CBA + BiL respectively. As shown in Table 4, CBA + BiLSTM achieved Acc = 76.66% compared to Acc = 73.68% for CBA + CNN, an improvement of 2.98%. the CBA + BiLSTM achieved AUROC = 0.8148 and AUPRC = 0.8192, corresponding to improvements of 0.19% and 0.52% over CBA + CNN. BiLSTM processed CGR features more effectively than CNN, though the performance difference between the two architectures is moderate. Based on these findings, we selected CBA + BiLSTM for integration into the multi-view architecture, as it showed marginally better overall performance across most evaluation metrics.

**Table 4:** Performance comparison of dual-branch architectures with different branch 2 configurations

Classifiers	AUROC	Acc (%)	Sn (%)	Sp (%)	MCC (%)	Pre (%)	F1 (%)	AUPRC
CBA/CNN	0.8129	73.68	74.46	**79.66**	**49.22**	73.79	**74.13**	0.814
CBA/BiL	**0.8148**	**76.66**	**74.54**	78.72	49.12	**74.55**	73.81	**0.8192**

Bold values represent the best results

### Impact of SimCLR contrastive learning integration and branch-wise ablation analysis

We performed ablation experiments on four configurations. Table 5 presents the performance comparison across four configurations: (1) dual-branch without contrastive learning (CBA + BiL), (2) dual-branch with contrastive learning S(CBA + BiL), (3) branch 1 only with contrastive learning S(CBA), and (4) branch 2 only with contrastive learning S(BiL).

**Table 5:** Performance comparison of contrastive learning Integration and branch-wise ablation

Classifiers	AUROC	Acc (%)	Sn (%)	Sp (%)	MCC (%)	Pre (%)	F1 (%)	AUPRC
CBA + BiL	0.8148	72.19	73.71	75.42	47.60	72.96	73.34	0.8080
S(CBA + BiL)	**0.8189**	**74.97**	**76.28**	**77.52**	**52.55**	**75.65**	**75.96**	**0.8270**
S(CBA)	0.7969	72.19	73.63	75.37	47.57	72.92	73.27	0.8094
S(BiL)	0.6851	71.10	62.31	54.75	26.36	64.69	63.48	0.6948

Bold values represent the best results

Adding contrastive learning to the dual-branch architecture (S(CBA + BiL)) improved Acc from 72.19% to 74.97% and MCC from 47.60% to 52.55% compared to CBA + BiL. AUROC increased from 0.8148 to 0.8189 (0.41% improvement), and AUPRC from 0.8080 to 0.8270 (1.90% improvement). These improvements validate the effectiveness of contrastive learning in enhancing feature discrimination.

Single-branch Analysis. When examining individual branches under the contrastive learning framework, S(CBA) achieved an AUROC of 0.7969 and AUPRC of 0.8094, demonstrating that the DNABERT-processed sequential features through CNN-BiLSTM-Attention maintain strong predictive capability. However, S(BiL) showed significantly lower performance with AUROC of 0.6851 and AUPRC of 0.6948, indicating limited performance from branch 2 alone.

Complementary Feature Fusion: The performance gap between S(CBA + BiL) and individual branches highlights the synergistic effect of dual-branch fusion. S(CBA + BiL) outperforms S(CBA) by 2.20% in AUROC and 1.76% in AUPRC, while surpassing S(BiL) by 13.38% in AUROC and 13.22% in AUPRC. Notably, the MCC improvement is substantial: S(CBA + BiL) achieves 52.55%, compared to 47.57% for S(CBA) and only 26.36% for S(BiL). This 4.98% improvement over S(CBA) and 26.19% over S(BiL) in MCC demonstrates that the dual-branch architecture effectively captures complementary information that neither branch can fully capture independently.

The ablation results confirm that while branch 1 serves as the primary feature extractor, branch 2 provides crucial supplementary structural information that enhances overall model performance. The contrastive learning framework further amplifies this complementarity by learning invariant representations across augmented views, resulting in the superior performance of the complete S(CBA + BiL) architecture.

### Comparison with other different feature encoding methods

Besides, the following content compared the prediction performance of the three feature encoding methods. The experiment encoded the sequences by our DNABERT and the two commonly used schemes, BERT, and word2vec, respectively, then applied the same CBA, CBA + BiL, and S(CBA + BiL) model to predict the modification site based on the same train dataset.

Table 6 compares the performance of three feature encoding methods (BERT, word2vec, and DNABERT) under the CBA architecture. Table 7 presents the results when these encodings are applied to the CBA + BiL model. Table 8 shows the performance after incorporating SimCLR contrastive learning into the CBA + BiL.

**Table 6:** Feature encoding comparison under CBA model

Classifiers	AUROC	Acc (%)	Sn (%)	Sp (%)	MCC (%)	Pre (%)	F1 (%)	AUPRC
CBA_BERT_	0.78	70.90	72.31	**74.05**	44.86	71.57	71.94	0.7935
CBA_Word2vec_	0.7919	67.80	72.47	77.53	45.64	70.48	71.47	0.8032
CBA_DNABERT_	**0.8105**	**72.33**	**72.89**	73.25	**45.64**	**72.67**	**72.78**	**0.8041**

Bold values represent the best results

**Table 7:** Feature encoding comparison under CBA + BiL model

Classifiers	AUROC	Acc (%)	Sn (%)	Sp (%)	MCC (%)	Pre (%)	F1 (%)	AUPRC
CBA + BiL_BERT_	0.791	72.12	**74.04**	76.22	**48.54**	**73.06**	**73.55**	0.7986
CBA + BiL_Word2vec_	0.8021	69.30	73.55	**77.87**	47.47	71.69	72.61	0.8031
CBA + BiL_DNABERT_	**0.8148**	**72.19**	73.71	75.42	47.60	72.96	73.34	**0.8080**

Bold values represent the best results

**Table 8:** Feature encoding comparison under S(CBA + BiL) model

Classifiers	AUROC	Acc (%)	Sn (%)	Sp (%)	MCC (%)	Pre (%)	F1 (%)	AUPRC
S (CBA + BiL)_BERT_	0.7792	72.83	71.98	70.68	44.77	72.47	72.22	0.7879
S (CBA + BiL)_Word2vec_	0.7756	71.64	71.98	72.52	44.55	71.8	71.89	0.7861
S (CBA + BiL)_DNABERT_	**0.8189**	**74.97**	**76.28**	**77.52**	**52.55**	**75.65**	**75.96**	**0.8270**

Bold values represent the best results

Across all three architectures, DNABERT-based encoding consistently outperformed BERT-based and word2vec-based. In the single-pathway CBA model (Table 6), DNABERT-based achieved 72.33% Acc compared to 70.9% for BERT-based and 67.8% for word2vec-based. The dual-branch CBA + BiL architecture (Table 7) amplified these differences, with DNABERT-based reaching 72.19% Acc while BERT-based and word2vec-based achieved 72.12% and 69.3%, respectively. The introduction of contrastive learning in S(CBA + BiL) (Table 8) further widened the performance gap, with DNABERT-based achieving 74.97% Acc compared to 72.83% for BERT-based and 71.64% for word2vec-based.

The dual-branch architecture (CBA + BiL) improved performance in the vast majority of evaluation metrics across three different encoding methods compared to the single-pathway CBA, indicating that parallel feature processing through different network structures captures complementary information. However, the benefit in AUROC varied by encoding method: word2vec-based showed a 0.1% improvement (0.7919 to 0.8021), while DNABERT-based gained 0.43% (0.8105 to 0.8148). When contrastive learning was applied, the AUROC of the DNABERT-based model improved by 0.41%, while word2vec-based actually decreased by 2.65%. This suggests that domain-specific pre-training in DNABERT provides representations better suited for learning from augmented views, whereas static word embeddings lack the adaptability required for contrastive learning frameworks.Correspondingly, the DNABERT-based model achieved 3.97% higher Acc than the BERT-based model, indicating that domain-specific pre-training on biological sequences provides better feature representations than general-purpose language models for this task.

### Comparison with state-of-the-art approaches

**Fig. 2:**
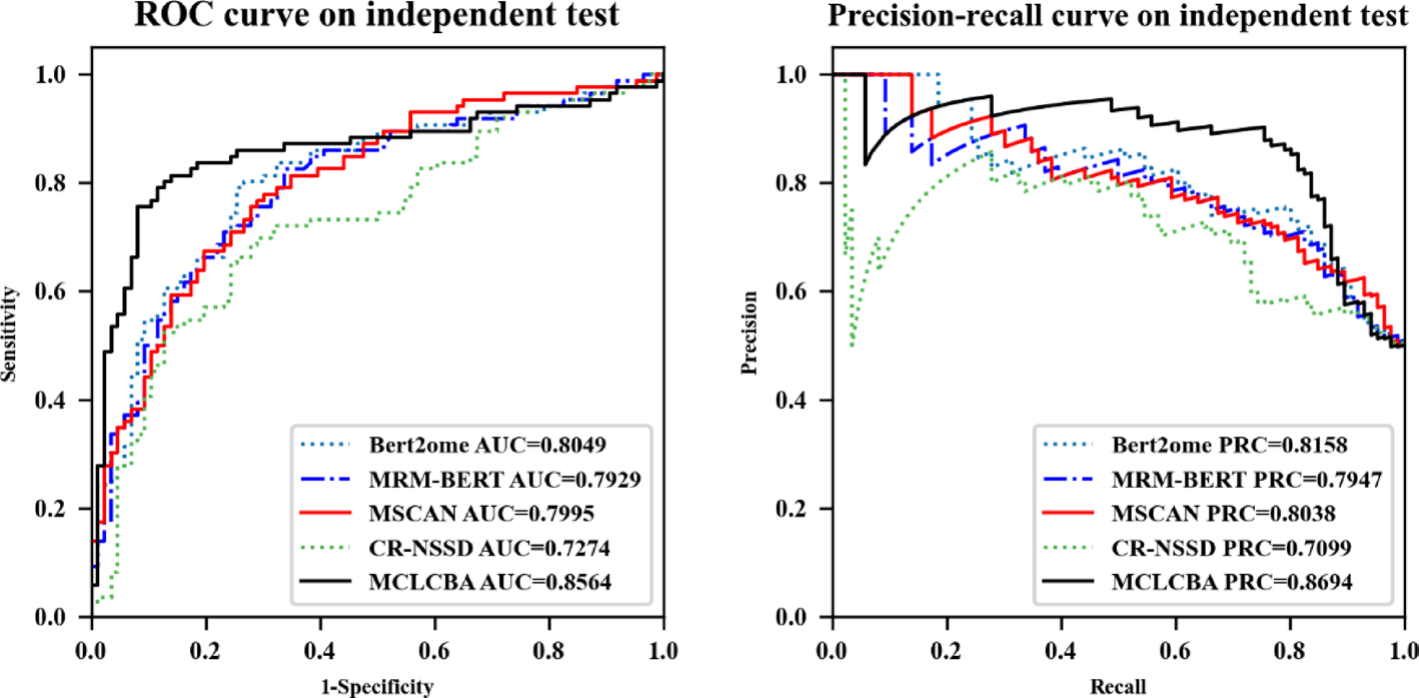
The ROC and PRC of MCLCBA and other state-of-the-art models on the test set

Experimental results on the independent m^7^G test dataset show MCLCBA’s performance over the baseline algorithms, as shown in Fig. 2. The MCLCBA ROC curve is generally located above those of the other baseline algorithms, demonstrating better discriminative capability. Specifically, MCLCBA achieved AUROC 0.8564, which represents improvements of 5.15, 6.35, 5.69, and 12.9% compared to Bert2ome, MRM-BERT, MSCAN, and CR-NSSD, respectively. From the PR curves, MCLCBA’s curve is consistently above those of other algorithms, achieving an AUPRC of 0.8694, representing improvements of 5.36, 7.47, 6.56, and 15.95% compared to Bert2ome, MRM-BERT, MSCAN, and CR-NSSD, respectively. The ROC curve of MCLCBA is closer to the top-left corner, while the PR curve is closer to the top-right corner, indicating that the model achieves high precision while maintaining high recall, demonstrating excellent overall performance. Through DNABERT and CGR multi-view feature extraction and contrastive learning mechanisms, MCLCBA can learn more discriminative feature representations under conditions of limited sample size.

Table 9 presents a detailed performance metric comparison of each algorithm on the m^7^G independent test set. MCLCBA achieved the highest Acc of 80.81%, outperforming the second-best algorithm Bert2ome by approximately 5%. In terms of the Sn metric, MCLCBA achieved the highest value of 83.72%, exceeding Bert2ome by 2.32% and indicating superior capability in correctly identifying true modification sites, thereby effectively reducing false negative rates. In terms of the Pre metric, MCLCBA reached the highest value of 79.12%, surpassing the next-best algorithm MRM-BERT by 3.48%. Notably, MCLCBA achieved the highest MCC value of 61.73%, significantly outperforming all other algorithms, with a 10.22% improvement over the next-best algorithm Bert2ome.

**Table 9:** Performance comparison of model on the independent m^7^G test set

Classifiers	AUROC	Acc (%)	Sn (%)	Sp (%)	MCC (%)	Pre (%)	F1 (%)	AUPRC
Bert2ome	0.8049	75.58	81.4	69.77	51.51	72.92	76.93	0.8158
MRM-BERT	0.7927	73.26	68.6	77.91	46.71	75.64	71.95	0.7947
MSCAN	0.7995	73.83	77.91	69.77	47.83	72.04	74.86	0.8038
CR-NSSD	0.7274	69.77	72.09	67.44	39.58	68.89	70.45	0.7099
MCLCBA	**0.8564**	**80.81**	**83.72**	**77.91**	**61.73**	**79.12**	**81.36**	**0.8694**

Bold values represent the best results

**Fig. 3:**
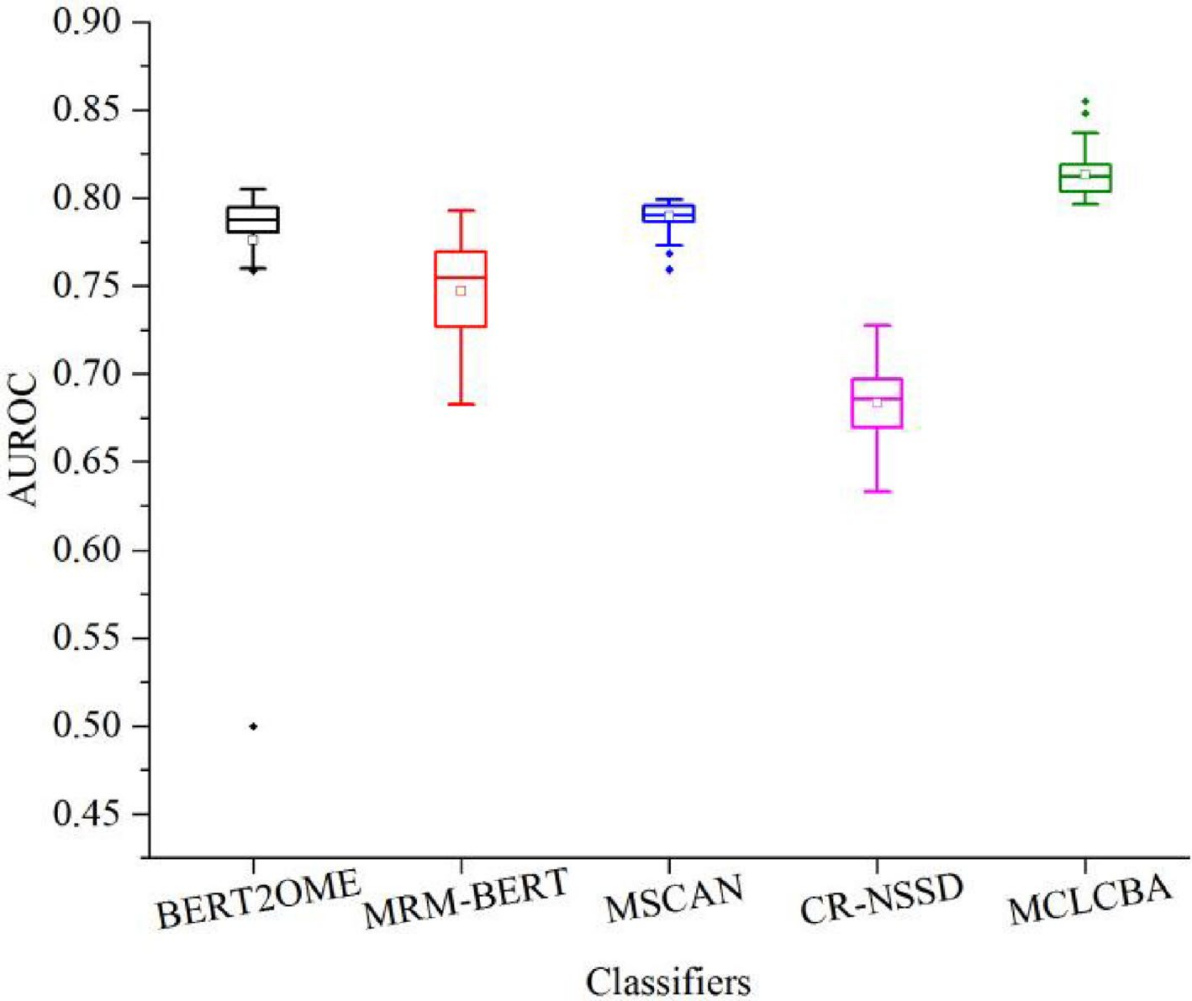
Box plots of AUROC distributions for MCLCBA and baseline algorithms over 100 independent runs

To assess the stability and reproducibility of MCLCBA and its comparison algorithms’ performance. Based on the m^7^A independent test set, this study conducted 100 independent repeated trials on the five algorithms. Boxplot analysis (Fig. 3) demonstrates that MCLCBA achieved not only the highest median AUROC but also the narrowest interquartile range and smallest overall distribution span. The significantly lower performance variability of MCLCBA compared to baseline algorithms is critical for reliable model deployment in real-world scenarios.

To comprehensively evaluate the generalization capability of MCLCBA across varying sample sizes, we systematically evaluated MCLCBA’s generalization capability across nine different RNA modification types using publicly available datasets from Song et al., ranging from small datasets (Gm with 636 positive samples) to large datasets (m^6^A with 41,307 positive samples). As summarized in Table 10, MCLCBA consistently outperformed all baseline algorithms in AUROC across every RM type.

**Table 10:** Comparative AUROC performance on independent test sets for nine RM types

Classifiers	m^6^Am	Am	Cm	Gm	Um	m^5^C	m^5^U	m^1^A	m^6^A
Bert2ome	0.9867	0.9220	0.8985	0.9606	0.9011	0.9644	0.9623	0.8383	0.9819
MRM-BERT	0.9854	0.8777	0.8741	0.9344	0.8447	0.9694	0.9737	0.8390	0.9799
MSCAN	0.9905	0.9292	0.9204	0.9578	0.8967	0.9730	0.9634	0.8541	0.9834
CR-NSSD	0.9587	0.8380	0.8011	0.8216	0.7494	0.9137	0.8698	0.7884	0.9764
MCLCBA	**0.9911**	**0.9430**	**0.9395**	**0.9770**	**0.9071**	**0.9760**	**0.9777**	**0.8598**	**0.9838**

Bold values represent the best results

### Interpretability analysis of learned features

We visualized learned features using the m7G test dataset on the m^7^G test dataset.

**Fig. 4:**
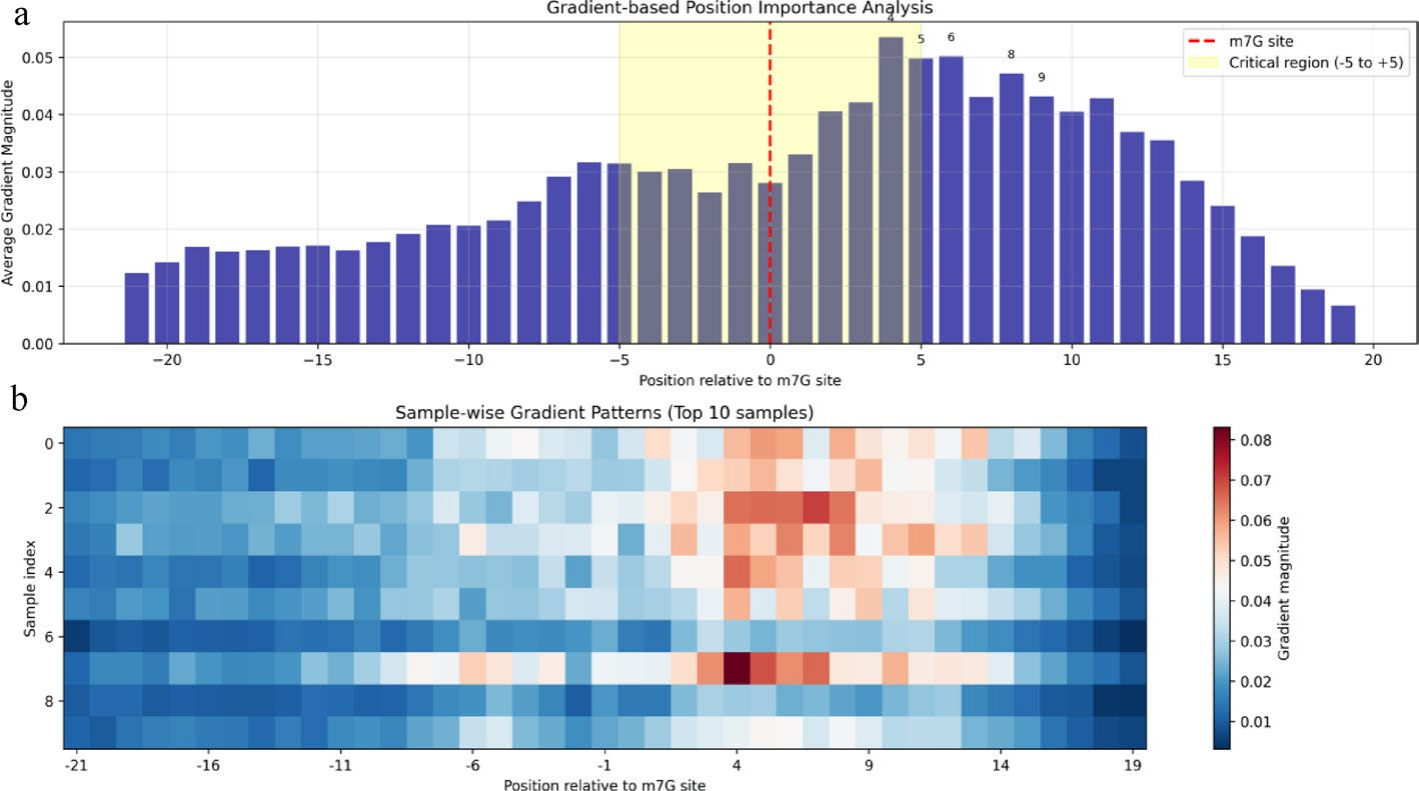
Gradient-based position importance analysis for m^7^G site prediction. **a** Average gradient magnitude at each position relative to the m^7^G site, with the critical region (− 5 to + 5) highlighted in yellow. **b** Sample-wise gradient patterns across 10 representative test samples showing consistent focus on the modification site vicinity

Gradient-based importance analysis revealed that the model focuses on positions surrounding the m^7^G modification site, with positions + 4 to + 9 showing peak importance values (Fig. 4a). The region from − 5 to + 5 demonstrated substantially higher gradient magnitudes compared to flanking sequences, indicating focus on local sequence context. The sample-wise gradient heatmap showed consistent patterns across multiple test samples (Fig. 4b), indicating robust learning of position-specific features.

**Fig. 5:**
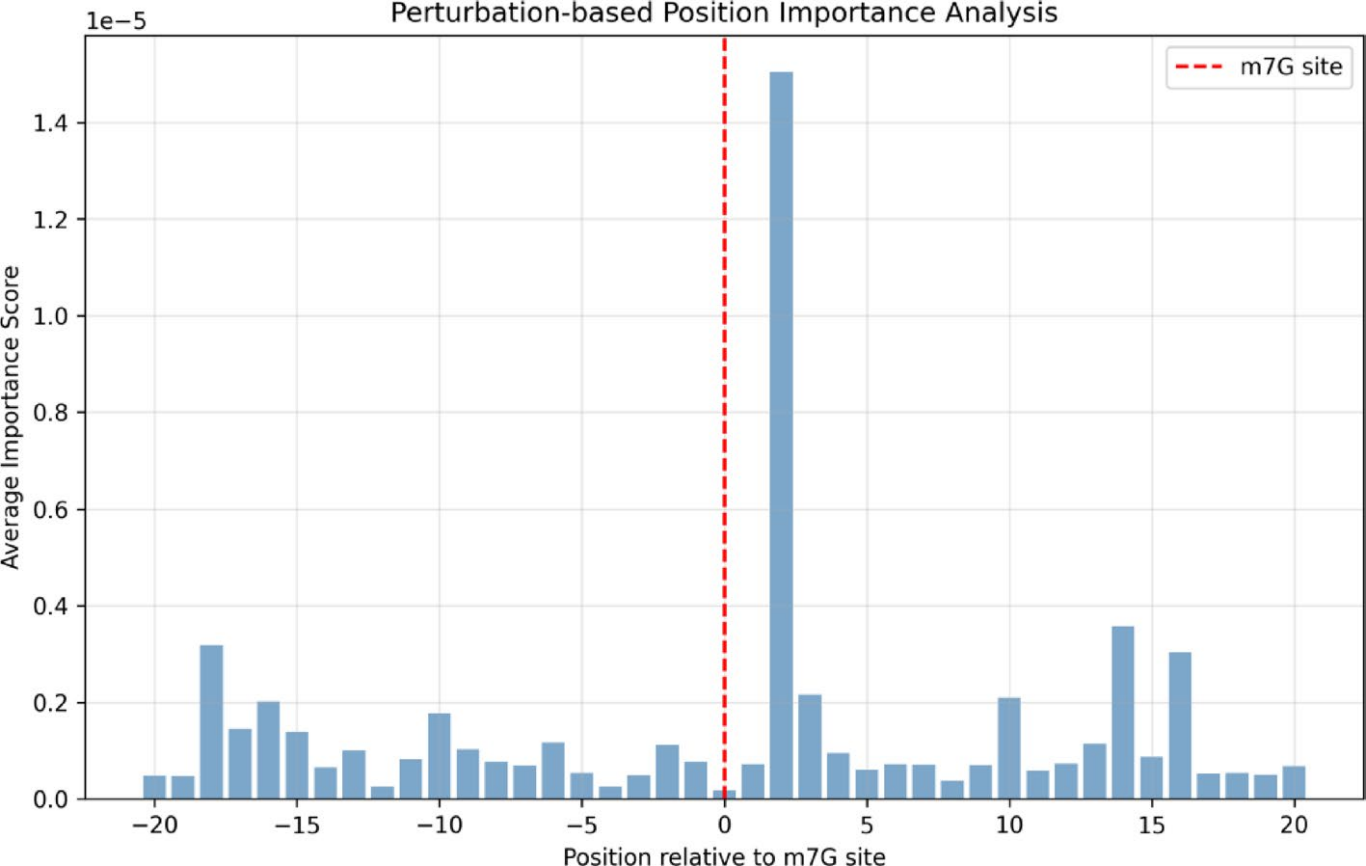
Perturbation-based position importance analysis revealing critical positions for m^7^G site recognition

Perturbation analysis identified position + 2 as the most critical for prediction accuracy (importance score: 1.5 × 10⁻^5^), exceeding even the modification site itself (Fig. 5). This finding suggests that downstream sequence context plays a crucial role in m^7^G site recognition. Additionally, peaks at positions − 18, + 14, and + 16 indicate the model captures both local and distant sequence dependencies.

**Fig. 6:**
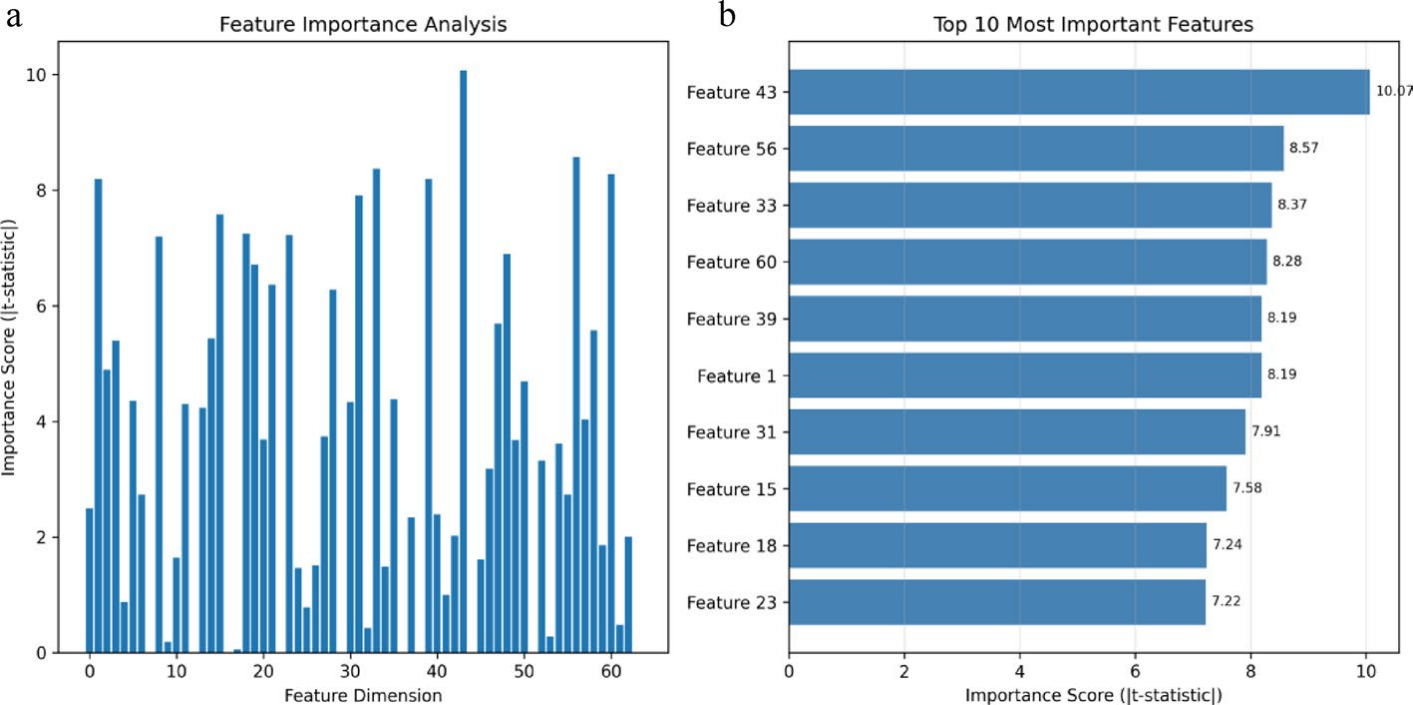
Feature importance analysis of learned representations in MCLCBA. **a** Distribution of t-statistic values across 64-dimensional feature space comparing positive and negative samples. **b** Top 10 features with highest discriminative power

Feature importance analysis of the 64-dimensional learned representations revealed strong discriminative power (Fig. 6a), with six features achieving |t-statistic| values exceeding 8. The top 10 most important features are shown in Fig. 6b, with Feature 43 reaching the highest t-statistic of 10.07. These high-importance features demonstrate that MCLCBA has learned robust internal representations for distinguishing m^7^G-modified from unmodified sequences.

These analyses show that MCLCBA captures position-specific sequence patterns and learns discriminative features relevant to m^7^G modifications, supporting our computational findings that the model captures conserved sequence features underlying RNA epigenetic modifications.

### Comparison with cross-modification validation approaches

**Fig. 7:**
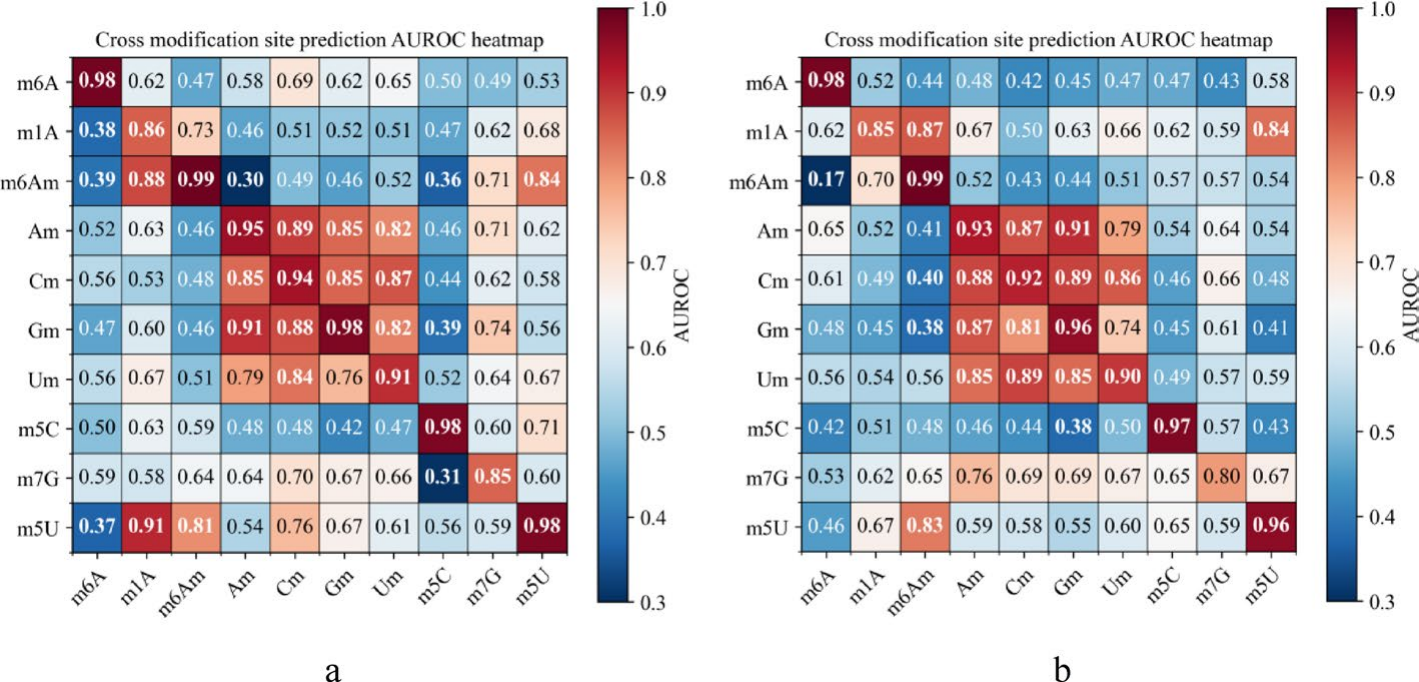
Heatmaps of AUROC values in cross-methylation validation. **a** MCLCBA, **b** MSCAN

We investigated MCLCBA’s capability for cross-modification tasks base on the 10 types RNA methylation dataset by Song et al.. Comparative analysis with MSCAN demonstrated MCLCBA’s superior performance in cross-modification prediction. We selected MSCAN as the primary benchmark for two reasons: first, among the ten methods evaluated by Song et al., MSCAN achieved the second-highest performance, making it the most relevant state-of-the-art baseline; second, the cross-validation framework using these ten RM-type datasets was originally established in the MSCAN study, ensuring methodological consistency and direct comparability. Heatmaps of AUROC values for cross-RM validation are displayed in Fig. 7. Horizontal axis represents the 10 RM types used for model training.Vertical axis denotes the 10 RM types used for performance testing.Color intensity represents the AUROC values (darker hues indicate higher values). Notably, the diagonal elements of MCLCBA (same-type prediction) reached a minimum of 0.85, exceeding MSCAN’s 0.8 by 5%. For Nm-type modifications (Am, Cm, Gm, Um), MCLCBA maintained a minimum cross-prediction AUROC of 0.76, surpassing MSCAN’s 0.74 by 2%. MCLCBA’s consistent superiority across technical dimensions provides empirical validation for the efficacy of its framework.For technical validation under limited sample size, MCLCBA achieved an average AUROC of 0.6355 when trained exclusively on m^7^G data to predict nine other modification types, outperforming MSCAN (0.5811) with a 5.44% improvement. This result validates MCLCBA’s capability to learn transferable features from limited data and demonstrates the efficacy of its multi-view contrastive learning framework in extracting biologically relevant patterns from limited data.Regarding overall cross-modification performance, the mean off-diagonal AUROC for MCLCBA reached 0.6067, exceeding MSCAN’s 0.5894 by 1.73%. Although this improvement is relatively modest, the average improvement in metrics reflects the superior feature generalization capacity inherent to contrastive learning. The contrastive learning mechanism facilitates learning of general feature representations invariant to diverse modification types.

## Discussion

MCLCBA combines multi-view learning with contrastive training for RNA methylation site prediction. On the m^7^G dataset (1210 samples), MCLCBA achieved AUROC of 0.8564, compared to 0.8049 for Bert2ome and 0.7995 for MSCAN. The performance gain comes from integrating DNABERT and CGR encodings through parallel branches with contrastive learning optimization.

DNABERT-6 and CGR encodings provide different sequence representations. DNABERT-6 captures local sequence motifs and long-range contextual dependencies through 6-mer tokenization, as shown by Zhang et al. [40]. CGR encodes sequences through geometric transformation. Combining these features improved prediction accuracy.

The CBA architecture processes sequences at multiple scales: CNN detects local motifs, BiLSTM models long-range dependencies, and attention weights feature importance by position.

SimCLR contrastive learning improved generalization. With limited samples, contrastive learning enforces robust and transferable feature representations by maximizing intra-class similarity within positive sample pairs while minimizing inter-class similarity for negative pairs. Similar sequences tend to have similar modification patterns. Yang et al. [41] showed similar results in gene classification.

Cross-modification tests showed relationships between methylation types. Am, Cm, Gm, and Um modifications showed cross-prediction ability, likely due to shared chemical properties. The structural analyses conducted by Wang et al. [42] provide a theoretical foundation for understanding these shared molecular recognition patterns. The m^7^G-trained models achieve substantial cross-prediction performance on heterologous modifications (mean AUROC = 0.6355), suggesting shared sequence features. MCLCBA may capture features common to different methylation types.

## Conclusions

We developed MCLCBA for RNA methylation site prediction with limited samples. MCLCBA uses multi-view contrastive learning architecture, integrating DNABERT and CGR feature representations to learn discriminative and transferable features from limited datasets. MCLCBA improved upon existing methods in both single and cross-modification prediction tasks. The method performed well on sample-limited m^7^G datasets and generalized across nine methylation site datasets. This approach may be useful for studying RNA methylation.

Several limitations remain that point to promising avenues for continued research. First, while our current CGR feature extraction approach demonstrates effectiveness, this work represents an initial step in a broader research program. We plan to systematically explore alternative encoding methods, to identify optimal strategies for different application contexts. Second, negative sampling optimization in contrastive learning requires enhancement, particularly for highly imbalanced datasets. Third, this study is limited to datasets with a limited number of samples. Due to the lack of recognized dataset for few-shot learning, the method has not yet extended to few-shot learning. However, it is worth looking forward to testing the performance of MCLCBA if dataset for few-shot learning become available in the future.

## Materials and methods

### Datasets

We used RNA modification datasets from Song et al. [43]. The dataset encompasses 12 RNA modification types, of which 10 are RNA methylation modifications (m^6^A, m^1^A, m^5^C, m^5^U, m^6^Am, m^7^G, Am, Cm, Gm, and Um) and 2 are non-methylation modifications (Ψ and I). As we focus on RNA methylation, we selected the 10 RNA methylation modification types for analysis (Table 1), which are available for download at http://47.242.23.141/MCLCBA/index.php. All datasets maintain balanced positive-to-negative sample ratios of 1:1 with standardized sequence lengths of 41 nucleotides. Sequences with original lengths below 41 nucleotides underwent padding augmentation by appending gap characters (“–”) at terminal positions. Given the m^7^G dataset contains the most constrained sample size (605 training positives, 86 testing positives), we designated it as the primary training set for hyper-parameter optimization, while the remaining nine datasets served for evaluating model generalization capability.

### Feature encoding representation

We used DNABERT and CGR for feature encoding. DNABERT is a DNA sequence pre-training model based on the BERT architecture for genomic sequence analysis. The implementation utilizes k-mer tokenization strategies to construct sequence vocabularies, with four model variants trained for k ∈ (3, 4, 5, 6). Each model’s vocabulary encompasses 4^k^ possible k-mer combinations plus five specialized tokens: [CLS], [PAD], [UNK], [SEP], and [MASK]. The model applies random masking to approximately 15% of input k-mer tokens and learns nucleotide sequence statistics and contextual dependencies by minimizing the Masked Language Model (MLM) objective for masked token prediction. Through Transformer self-attention mechanisms, DNABERT captures long-range positional dependencies within sequences, generating high-dimensional feature contextually enriched representations. This capability proves particularly valuable for identifying RNA modification sites exhibiting complex motif patterns.

CGR maps 1D nucleic acid sequences to 2D space using fractal geometry. CGR preserves both base type information and positional relationships. The algorithm effectively encodes sequence geometric structural information through the iterative mapping process, ensuring that the final coordinates of each nucleotide in the 2D space simultaneously carry two types of information: the base type characteristics of the current nucleotide and its geometric positional relationships within the sequence. Due to the mathematical properties of iterative mapping, the contribution weights of nucleotides at different sequence positions to the calculation of the current coordinates exhibit a decreasing distribution pattern, i.e., nucleotides closer to the current position exert stronger influence, while the influence of more distant positions decays exponentially. This weight distribution mechanism ensures that the CGR representation effectively integrates global sequence pattern information while maintaining sensitivity to local sequence features.

In implementation, the CGR method establishes a standardized mapping relationships between nucleotides and vertices of a 2D unit square, where A, C, G, T correspond to the four vertices with coordinates (0, 0), (0, 1), (1, 1), and (1, 0) respectively. Each nucleotide’s coordinates depend on both the previous nucleotide’s coordinates and its own base type. For a given RNA sequence ${\mathbf{X}}_{0} = \left\{ {x^{1} ,x^{2} , \cdots ,x^{N} } \right\}$, geometric coordinates are computed via an iterated function system (IFS):

7 $$ p_{i} = p_{i - 1} + \alpha (v_{{x^{i} }} - p_{i - 1} ) $$

where the scaling factor $\alpha$ = 0.5 ensures convergence and non-overlapping mappings. Let $p_{i} \in R^{2}$ denote the 2D coordinates of the *i-*th nucleotide, initialized at the square centroid $p_{0} = (0.5,0.5)$, and $v_{{x^{i} }}$ the vertex coordinate of the corresponding nucleotide. To ensure compatibility with DL model input specifications, we transform 2D coordinates into a one-dimensional feature vector. For each coordinate, we calculate a scalar state value via the difference between its vertical and horizontal components:

8 $$ State_{i} = p_{i}^{y} - p_{i}^{x} $$

In the above equation, $p_{i}^{y}$ and $p_{i}^{x}$ denote the horizontal and vertical coordinates of respectively. The ordered sequence of these scalar values Statei then forms the final 1D feature vector that represents the original nucleotide sequence. Combining (7) and (8), we can obtain a sequence of nucleotide states by strictly maintaining the temporal sequential dependencies in **X**_0。_

The main input features are encoded using DNABERT and denoted **X**, while the supplementary input features are encoded using CGR and denoted ${\mathbf{X}}_{extra}$. In the main input feature module, each sequence of length $L_{0}$ is first divided into ($L_{0} - k + 1$) k-mers using a sliding window (with a stride of 1). Each sequence is then augmented with special tokens [CLS] and [SEP], resulting in a final length of ($L_{0} - k + 3$). Input words are converted into token representations based on the vocabulary dictionary, and sequences are represented as the embedding matrix ${\mathbf{X}} \in {\mathbb{R}}^{{(L_{0} - k + 3) \times D}}$, where *D* = 768 denotes the word vector dimension, as shown in Fig. 1A. In the supplementary input feature module, each sequence is represented as the feature matrix ${\mathbf{X}}_{extra} \in {\mathbb{R}}^{{L_{0} \times D}}$, where *D* = 1 denotes the feature dimension of CGR encoding, as shown in Fig. 1B.

#### Multi-view contrastive learning model

This study designed a multi-view contrastive learning framework that guides models to learn highly discriminative feature representations through positive–negative sample pair construction and representation learning objective optimization. The core principle of this framework involves maximizing cosine similarity between representation vectors of intra-class samples while minimizing cosine similarity between inter-class representation vectors, enabling models to establish compact and well-separated feature distributions within the projection space. As illustrated in Fig. 1, the model architecture comprises four essential components: data augmentation, multi-view encoder, projection head, and contrastive loss function, corresponding to parts Fig. 1C–F.

#### Data augmentation

Following the contrastive learning paradigm established in SimCLR [39], we employ a stochastic data augmentation module. For each original RNA sequence sample x, we generate two correlated views to form a positive pair ($\tilde{x}_{q}$ and $\tilde{x}_{k}$) for subsequent contrastive learning. As shown in Fig. 1C, each sample *x* is initially represented by a multi-view feature set, comprising **X** and ${\mathbf{X}}_{extra}$. Two separate augmentation operators *t* and *t′* are independently sampled from a predefined family of augmentations $\Gamma$. Critically, each operator is applied to the entire multi-view feature set, but utilizes a distinct augmentation strategy tailored to each feature type within the set. Specifically, for the discrete primary input features extracted by DNABERT, this study employs random masking techniques for data augmentation. By generating a random tensor following a uniform distribution over the interval [0, 1] and comparing it with the predefined threshold $p_{mask}$, a binary mask matrix is constructed:

9 $$ M_{q} = \left\{ {\begin{array}{*{20}l} 1 \hfill & {\quad if\;U(0,1) > p_{mask} } \hfill \\ 0 \hfill & {\quad otherwise} \hfill \\ \end{array} } \right. $$

where U(0,1) denotes the uniform distribution over the interval [0,1]. This mechanism randomly masks approximately $p_{mask}$ of the feature elements, and augmented sequence features are obtained through element-wise multiplication:

10 $$ {\mathbf{X}}_{aug\_q} = {\mathbf{X}} \odot cast(M_{q} ,dtype({\mathbf{X}})) $$

where ⊙ denotes the Hadamard product (element-wise multiplication). The technical principle of this masking strategy lies in randomly obscuring partial feature elements to promote the model to learn robust feature representations that do not depend on specific positional information. For the continuous supplementary input features extracted by CGR, we employ an additive Gaussian noise strategy for data augmentation. This approach enhances feature diversity by introducing small-amplitude random perturbations:

11 $$ {\mathbf{X}}_{extra\_aug\_q} = {\mathbf{X}}_{extra} + N{(0,1)} \cdot \eta_{q} $$

where *N*(0,1) represents the standard normal distribution, and $\eta_{q}$ serves as the noise scale coefficient. The setting of this coefficient requires a balance between introducing sufficient randomness and preserving the integrity of the original features, ensuring that the augmented features retain the original geometric positional relationships.

To determine the optimal values for the masking ratio $p_{mask}$ and the noise magnitude $\eta_{q}$, we conducted a grid search over a range of possible values. Specifically, we evaluated $p_{mask}$ in the set [0.05,0.10,0.15,0.20,0.25] and $\eta_{q}$ in the set [0.005,0.01,0.02,0.03,0.04]. The experiments were conducted on the training dataset. We observed that the combination of $p_{mask} = 0.15$ and $\eta_{q} = 0.01$ yielded the highest AUC on the validation set. These values were subsequently used in all main experiments.

#### Multi-view encoder

The encoder $f_{\theta } \left( \cdot \right)$ processes augmented samples through two parallel branches to generate feature representations $h_{q}$ and $h_{k}$ for the Query and Key pairs. As shown in Fig. 1D, this architecture can simultaneously process context features based on DNABERT and geometric structural features based on CGR, achieving effective integration of multi-dimensional feature information.

The primary input branch extracts deep contextual association features of RNA sequences, including complex sequence patterns such as long-range dependencies. This branch adopts a cascaded architecture of CNN, BiLSTM, and attention mechanisms, constructing a complete processing pipeline from local feature extraction to global information integration. The CNN layer first applies a one-dimensional convolution transformation to the input DNABERT features, identifying local sequence patterns. The ReLU activation function provides nonlinear transformation capability, enhancing the model’s ability to represent complex patterns. We apply batch normalization and dropout operations after the convolutional layer to improve training stability. Since identifying RNA modification sites depends on both local sequence patterns and upstream/downstream sequences, we process the data through a BiLSTM to obtain bidirectional temporal features $h_{t}$, with the hidden unit dimension set to 64. To highlight the contribution of key positions to modification site identification, the network introduces an attention mechanism. The attention mechanism uses tanh function to calculate the importance score $e_{t}$ at each time step via learnable parameters $W_{a}$, and $b_{a}$, normalizes the scores into weights $a_{t}$ via softmax function, and finally generates a weighted context vector *c* through linear combination. Overall, the primary feature extraction branch builds a hierarchical pipeline comprising: (1) local feature extraction, (2) long-range dependency capture, and (3) important position emphasis. This constructs a contextual representation that enables deep understanding of RNA sequence environments.

The supplementary input branch extracts geometric structural features of RNA sequences, complementing feature dimensions potentially overlooked by the primary branch. Although CGR features already contain positional encoding information, sequential learning is still required to extract their intrinsic temporal dependencies. This branch employs BiLSTM to establish temporal associations between CGR encodings, enabling each position’s representation to integrate structural information from neighboring positions, with the hidden unit dimension also set to 64, establishing the foundation for subsequent cross-branch feature fusion.

#### Projection head

The projection head $g\left( \cdot \right)$ processes the concatenated features from both the branch 1 and branch 2, is a key component that maps high-dimensional feature representations to a lower-dimensional space optimized for the contrastive loss. As illustrated in Fig. 1E.

The core computational bottleneck in contrastive learning lies in calculating the pairwise similarity matrix for the entire batch. The complexity of this operation is given by:

12 $$ {\mathcal{O}}(B^{2} \cdot D) $$

where *B* is the batch size and *D* is the dimensionality of the concatenated representation from both branches.

The projection head $g\left( \cdot \right)$, typically implemented as a multi-layer perceptron (MLP) as defined in Eq. (13), introduces an additional computational overhead. The complexity of projecting the entire batch of features ${\mathbf{H}} \in {\mathbb{R}}^{B \times D}$ through the MLP is:

13 $$ {\mathcal{O}}(g({\mathbf{H}})) = {\mathcal{O}}(B \cdot D \cdot P) $$

where *P* is the output dimension of the projection head (*P* ≪ *D*).

After projection, as illustrated in Fig. 1E, the contrastive loss is calculated using the lower-dimensional representations $z_{q} \in {\mathbb{R}}^{P}$, reducing the complexity of the similarity computation from ${\mathcal{O}}(B^{2} \cdot D)$ to ${\mathcal{O}}(B^{2} \cdot P)$.

Therefore, the total computational overhead when using the projection head comprises two parts: the cost of the projection operation itself and the cost of calculating the contrastive loss on the projected features. This combined complexity is:

14 $$ {\mathcal{O}}_{total} = {\mathcal{O}}(B^{2} \cdot P) + {\mathcal{O}}(B \cdot D \cdot P) $$

Comparing this to the original cost of calculating loss on the high-dimensional features ${\mathcal{O}}(B^{2} \cdot D)$, we achieve significant efficiency gains when *P* ≪ *D* and *B* is large, as ${\mathcal{O}}(B^{2} \cdot P) + {\mathcal{O}}(B \cdot D \cdot P) \ll {\mathcal{O}}(B^{2} \cdot D)$.

Calculating the contrastive loss on the projected representations $z_{q}$ not only improves computational efficiency but also leads to superior learning performance. The projection head operates on the fused multi-view representation, enabling the contrastive learning framework to learn invariant features across both sequential (DNABERT) and structural (CGR) encoding modalities. This unified projection ensures that the contrastive loss optimizes representations that capture complementary information from both branches. After pre-training, the projection head $g\left( \cdot \right)$ is discarded, and only the encoder $f\left( \cdot \right)$ and the representation *h* are used for downstream tasks.

#### Contrastive loss function

We use the Normalized Temperature-scaled Cross Entropy loss (NT-Xent). Given a set $\left\{ {x_{i} } \right\}$ including a positive pair of examples $\tilde{x}_{q}$ and $\tilde{x}_{k}$, the contrastive prediction task aims to identify $\tilde{x}_{k}$ in $\left\{ {x_{i} } \right\}_{i \ne q}$ for a given $\tilde{x}_{q}$.We use NT-Xent as the optimization objective for contrastive learning. For a mini-batch of N samples, the data augmentation strategy generates 2*N*-augmented samples. Given a positive pair of examples $\tilde{x}_{q}$, and $\tilde{x}_{k}$, the remaining 2(*N* − 1) augmented samples are all treated as negative examples. The similarity between feature vectors is calculated using $\ell_{2}$ normalized cosine similarity.

15 $$ sim(\tilde{x}_{q} ,\tilde{x}_{k} ){ = }\frac{{\tilde{x}_{q}^{T} \tilde{x}_{k} }}{{||\tilde{x}_{q} || \cdot ||\tilde{x}_{k} ||}} $$

This similarity metric represents the dot product between the $\ell_{2}$ normalized vectors $u$ and $v$, i.e., the cosine similarity. Then, for the positive pair of examples $\tilde{x}_{q}$, and $\tilde{x}_{k}$, we define the contrastive loss function as:

16 $$ \ell_{q,k}^{cont} = - \log \frac{{\exp (sim(\tilde{x}_{q} ,\tilde{x}_{k} )/\tau )}}{{\sum\nolimits_{j = 1}^{2N} {{\mathbb{Z}}_{{\left[ {j \ne q} \right]}} \exp (sim(\tilde{x}_{q} ,\tilde{x}_{j} )/\tau )} }} $$

where: ${\mathbb{Z}}_{{\left[ {j \ne q} \right]}} \in \left\{ {0,1} \right\}$ is the indicator function, taking the value 1 only when $j \ne q$, and the temperature parameter $\tau$ controls the smoothness of the similarity distribution. Smaller $\tau$ values enhance the distinguishability between high-similarity sample pairs. For all positive sample pairs in the mini-batch, the final contrastive loss function we define as:

17 $$ {\mathcal{L}}^{cont} = \frac{1}{2N}\sum\nolimits_{k = 1}^{N} {\left[ {\ell_{2k - 1,2k}^{cont} + \ell_{2k,2k - 1}^{cont} } \right]} $$

In the specific implementation, the unit matrix labels = tf.eye(batch_size) is used to effectively label positive and negative pairs, ensuring that diagonal elements (positive sample pairs) are labeled as 1 and non-diagonal elements (negative pairs) are labeled as 0. This loss maximizes similarity between positive pairs while minimizing similarity to negative examples.

#### Unified multi-task learning objective

While the contrastive loss provides self-supervised regularization through representation learning, the complete MCLCBA framework extends beyond pure contrastive learning by incorporating supervised classification to directly optimize for methylation site prediction. This multi-task learning paradigm leverages the complementary strengths of both learning objectives to achieve superior performance in limited-data scenarios.

The supervised learning pathway processes the concatenated representation h through a dedicated classification branch. The classifier uses dense layers: 256 → 128 → 64 → 2 with ReLU activation and 0.1 dropout.

The classification objective employs categorical cross-entropy loss to optimize discrimination between methylated and non-methylated sites:

18 $$ {\mathcal{L}}^{cls} = - \frac{1}{N}\sum\limits_{i = 1}^{N} {\sum\limits_{c = 1}^{2} {y_{ic} } } \log (\hat{y}_{ic} ) $$

where *N* denotes the batch size, $y_{ic}$ represents the one-hot encoded ground truth label for sample i and class c, and $\hat{y}_{ic}$ indicates the predicted probability from the classification branch.

The framework optimizes a unified objective that synergistically combines both learning paradigms:

19 $$ {\mathcal{L}}^{total} = {\mathcal{L}}^{cls} + \lambda * {\mathcal{L}}^{cont} $$

The weighting parameter λ balances the contribution of contrastive learning within the multi-task framework. Our implementation employs an adaptive weighting strategy that responds to training dynamics. Initially set to λ = 0.5, this parameter remains fixed during the first 10 epochs to establish stable feature representations. Subsequently, λ adapts based on validation performance trends analyzed through a 5-epoch sliding window. When validation Acc exhibits negative trends, indicating performance saturation, λ decreases to a minimum of 0.3, reducing the influence of contrastive regularization. Conversely, positive trends trigger increases up to 0.5, encouraging enhanced representation learning. This adaptive mechanism prevents the contrastive objective from dominating convergence while maintaining its regularization benefits throughout training.

The multi-task architecture facilitates bidirectional knowledge transfer between objectives. The contrastive loss encourages learning of invariant features across augmented views, establishing robust representations that generalize beyond specific sequence variations. Simultaneously, the classification loss ensures these representations remain discriminative for the target task. This synergistic design proves particularly effective for RNA methylation site prediction, where limited training data necessitates maximal extraction of learning signal from available samples. The contrastive component effectively augments the training signal through self-supervised learning, while the classification component maintains focus on the primary prediction objective.

#### Training strategy and regularization

The implementation of large-scale modules such as CNN, BiLSTM, and attention mechanisms necessitates careful consideration of regularization strategies, particularly when applied to datasets with limited sample sizes. Our approach integrates multiple regularization techniques within a unified training framework. Dropout regularization was applied differentially across the network architecture, with a rate of 0.3 following the convolutional and BiLSTM layers in the primary branch and 0.1 in the projection head’s dense layers. These parameters were determined through systematic validation set performance monitoring to achieve optimal balance between model capacity and generalization.

An early stopping criterion with a patience parameter of 15 epochs was implemented to prevent overfitting while ensuring adequate training convergence. This mechanism monitors validation Acc and restores model weights from the checkpoint demonstrating peak validation performance upon termination. The architecture further incorporates batch normalization following convolutional operations and group normalization with 4 groups after BiLSTM layers, which collectively contribute to training stability and mitigate internal covariate shift phenomena. The learning rate schedule employs a 5-epoch warm-up phase followed by exponential decay with a rate of 0.9, facilitating stable optimization dynamics throughout the training process.

Beyond these explicit regularization mechanisms, the contrastive learning framework inherently provides implicit regularization through its objective function structure. The requirement to maintain representational consistency across augmented views while discriminating between distinct samples constrains the model to learn features that capture fundamental biological patterns rather than sample-specific artifacts.

## Data Availability

We used RNA modification datasets from Song et al. All data used in this study were already publicly available in the GEO database, RMBase, and RADAR database. In GEO database, m^6^A data can be collected from GSE71154, GSE86336, GSE98623 and GSE63753; m^1^A: GSE97908, GSE102040, GSE90963, GSE97419 and GSE70485; m6Am: GSE122948, GSE78040 and GSE63753; 2′-O-methyladenosine (Am, Cm, Gm, Um): GSE90164; m^5^C: GSE122260; m^7^G: GSE112276; m^5^U: GSE109183. 2′-O-methyladenosine data was also collected from the RMBase database under 2′-O-Me[http://rna.sysu.edu.cn/rmbase/2-O-Methylation.php] tag. All processed sequence data is freely available on the MultiRM web server at www.xjtlu.edu.cn/biologicalsciences/multirm or the MCLCBA web server at http://47.242.23.141/MCLCBA/index.php.

## Abbreviations

RNA: Ribonucleic acid
RM: RNA methylation
mRNA: messenger RNA
rRNA: ribosomal RNA
tRNA: transfer RNA
lncRNA: long non-coding RNA
snRNA: small nuclear RNA
miRNA: microRNA
circRNA: circular RNA
m^1^A: N1-methyladenosine
m^6^A: N6-methyladenosine
Ψ: Pseudouridine
m^6^Am: N6,2′-O-dimethyl adenosine
Am: 2′-O-methyladenosine
Cm: 2′-O-methylcytidine
Gm: 2′-O-methylguanosine
Um: 2′-O-methyluridine
m^5^C: 5-Methylcytidine
m^7^G: 7-Methylguanosine
m^5^U: 5-Methyluridine
I: Inosine
CNN: Convolutional neural network
BiLSTM: Bidirectional long short-term memory
CBA: CNN+BiLSTM+Attention
RF: Random forest
ML: Machine learning
NLP: Natural language processing
DL: Deep learning
MCLCBA: Multi-view contrastive learning with CNN-BiLSTM- Attention
Sn: Sensitivity
Sp: Specificity
Acc: Accuracy
Pre: Precision
F1: F1-score
MCC: Matthews correlation coefficient
AUROC: Area under the receiver operating characteristic
AUPRC: Area under the precision–recall curve
ENAC: Enhanced nucleic acid composition
BE: Binary encoding
CKSNAP: Composition of k-spaced nucleotide pairs
KMFE: k-mer frequency encoding
SimCLR: Simple framework for contrastive learning of visual representations
EIIP: Electron-ion interaction pseudopotential
NCP: Nucleotide chemical property
AE: Adaptive embedding
BGRU: Bidirectional gated recurrent unit
NMSE: Neighboring methylation state encoding
RNAWE: RNA word embedding
CRE: Cross-domain reconstruction encoder
CGR: Chaos game representation
LLMs: Large language models
BERT: Bidirectional encoder representations from transformers
DNABERT: DNA bidirectional encoder representations from transformers
